# Animal model of *Mycoplasma fermentans* respiratory infection

**DOI:** 10.1186/1756-0500-6-9

**Published:** 2013-01-08

**Authors:** Antonio Yáñez, Azucena Martínez-Ramos, Teresa Calixto, Francisco Javier González-Matus, José Antonio Rivera-Tapia, Silvia Giono, Constantino Gil, Lilia Cedillo

**Affiliations:** 1 Laboratorio de Investigación en Microbiología Oral, Facultad de Estomatología, Benemérita Universidad Autónoma de Puebla, 31 Poniente 1304, Col. Volcanes, Puebla, Pue, 72410, México; 2 Laboratorio de Micoplasmas. Centro de Investigaciones en Ciencias Microbiológicas del Instituto de Ciencias de la Benemérita Universidad Autónoma de Puebla, Edificio 103-J, Ciudad Universitaria, Col. San Manuel, Puebla, Pue, 72570, México; 3 Laboratorio de Bacteriología Médica. Escuela Nacional de Ciencias Biológicas, Instituto Politécnico Nacional, Prolongación de Carpio y Plan de Ayala s/n. Col. Santo Tomás, México, D.F. 11340, México; 4 Centro de Detección Biomolecular, Benemérita universidad Autónoma de Puebla, Boulevard Valsequillo s/n. Ciudad Universitaria, Col. San Manuel, Puebla, Pue, 72570, México

**Keywords:** Animal model, Mycoplasma, Mycoplasma fermentans, Respiratory infection

## Abstract

**Background:**

*Mycoplasma fermentans* has been associated with respiratory, genitourinary tract infections and rheumatoid diseases but its role as pathogen is controversial. The purpose of this study was to probe that *Mycoplasma fermentans* is able to produce respiratory tract infection and migrate to several organs on an experimental infection model in hamsters. One hundred and twenty six hamsters were divided in six groups (A-F) of 21 hamsters each. Animals of groups A, B, C were intratracheally injected with one of the mycoplasma strains: *Mycoplasma fermentans* P 140 (wild strain), *Mycoplasma fermentans* PG 18 (type strain) or *Mycoplasma pneumoniae* Eaton strain. Groups D, E, F were the negative, media, and sham controls. Fragments of trachea, lungs, kidney, heart, brain and spleen were cultured and used for the histopathological study. U frequency test was used to compare recovery of mycoplasmas from organs.

**Results:**

Mycoplasmas were detected by culture and PCR. The three mycoplasma strains induced an interstitial pneumonia; they also migrated to several organs and persisted there for at least 50 days. *Mycoplasma fermentans* P 140 induced a more severe damage in lungs than *Mycoplasma fermentans* PG 18. *Mycoplasma pneumoniae* produced severe damage in lungs and renal damage.

**Conclusions:**

*Mycoplasma fermentans* induced a respiratory tract infection and persisted in different organs for several weeks in hamsters. This finding may help to explain the ability of *Mycoplasma fermentans* to induce pneumonia and chronic infectious diseases in humans.

## Background

*Mycoplasma fermentans* was first isolated from the lower genitourinary tract of humans in the early 1950’s. Reports in the 1970’s of *Mycoplasma fermentans* in joints of patients suffering rheumatoid arthritis (RA) raised expectations for its role as pathogen [1,2]. When AIDS appeared, interest about mycoplasmas increased, species of the Class Mollicutes *Mycoplasma fermentans, Mycoplasma pirum* and *Mycoplasma penetrans* were isolated from AIDS patients. Several researches thought that mycoplasmas may act as cofactors in HIV associated disease progression [3].

The role of mycoplasmas in RA is controversial. Early work suggesting a link between *Mycoplasma fermentans* and human RA was unconvincing because it was isolated in a small proportion of patients, the bacteria was rarely isolated from the genitourinary tract and there was no evidence that it could colonize other sites [2]. The advent of polymerase chain reaction (PCR) provided new insights. Thus by the use of PCR, *Mycoplasma fermentans* has been found in the throat, peripheral blood leucocytes and urine of HIV positive and HIV negative patients [4]. More evidence has accumulated recently to establish an important and emerging role for *Mycoplasma fermentans* as pathogen in human respiratory tract and rheumatic diseases [1,2].

Presence of *Mycoplasma fermentans* in throat of humans let us think about the possibility that the bacteria may cause respiratory tract infections and spread to several organs, colonize them and persist there for several weeks. We developed an animal model of respiratory tract infection produced by *Mycoplasma fermentans.* Syrian hamsters have several advantages over other laboratory animals for evaluating the role of *Mycoplasma fermentans* as pathogen that is the reason why they were chosen [5] Animal models are particularly useful when the pathogenic role of a microorganism is controversial.

The purpose of this study was to probe that *Mycoplasma fermentans* is able to produce a respiratory tract infection and migrate to several organs in hamsters.

## Methods

### Animals

One hundred and eighty six hamsters were included in the study. Sixty hamsters were used to determine the infecting dose and 126 animals to test the ability of *Mycoplasma* to colonize and induce damage in the respiratory tract and different organs.

Female and male hamsters weighing 100–200 g were feed with chow and water “ad libitum”. Humidity, temperature, lighting and ventilation were controlled during the experiment. Animals were cared according to the guide for the care and use of laboratory animals of the U.S Department of Health and Human Services and Mexican legislation for Care and Use of Laboratory Animals (NOM-062-ZOO-199). Animals were cared in Claude Bernard bioterio of the University. Dr. Carlos Escamilla Weinman is the person who presides the committee for use of laboratory animals in our university and he gave us the approval for this study (BCB/PR/002/2010). Analgesics and anesthetics were administered to animals in order to avoid suffering. A throat swab of each animal was cultured to check that they were not colonized by *Mycoplasma fermentans* before the experiment began.

### Strains

Three strains of mycoplasma were used in this study. *Mycoplasma fermentans* P 140 isolated in our laboratory from the respiratory tract of an asthmatic patient (12 passages in culture media). *Mycoplasma pneumoniae* Eaton strain and *Mycoplasma fermentans* PG 18 isolated from the genitourinary tract were kindly provided by Dr. Gail H. Cassell from the University of Alabama at Birmingham (number of passages in culture media unknown).

Strains were cultured in E media and kept frozen at -70°C until used.

### Determination of the infecting dose

Animals were injected with Ketamine 200 mg/ kg of weight and pentobarbital 65 mg/kg of weight previous to the inoculation.

Sixty hamsters were used to determine the infecting dose of mycoplasmas. Hamsters were divided in three groups of 18 animals each (group I to III) and a control group of 6 hamsters (group IV). Six hamsters of group I were injected intratracheally with 0.1 ml of a culture that contained 10^3^ CFU/ml of *Mycoplasma fermentans* P 140, six hamsters were injected with 0.1 ml of a culture that contained 10^6^ CFU of the same strain and the other six hamsters were injected with 0.1 ml of a culture that contained 10^9^ CFU of the same strain. The same procedure was repeated with the other strains *Mycoplasma fermentans* PG 18 and *Mycoplasma pneumoniae*.

Six hamsters of group IV were not injected with mycoplasmas neither ketofen and were considered negative controls.

The day that hamsters were injected with mycoplasmas was considered day 0. Two hamsters of each group were sacrificed on days 10, 20 and 40. Animals were sacrificed by an injection of an overdose of sodium pentobarbital. Two fragments of lung, trachea, kidney and brain were cut one fragment of each organ was deposited in 0.9 ml of E media, other two tenfold dilutions were done to determine the presence of mycoplasma in organs. The other fragment was fixed and stained with hematoxylin and eosin to do a histopathological study. The same procedure was performed for each dose and negative controls.

### Inoculation with mycoplasmas

Animals were intramuscularly injected with Ketamine (200 mg/kg of weight) and pentobarbital (65 mg/kg of weight). Under aseptic conditions hamsters were intratracheally injected using a syringe.

Animals were divided in six groups of 21 hamsters each (group A-F). Hamsters of group A were intratracheally injected with 0.1 ml of a culture that contained 10^6^ CFU/ml of *Mycoplasma fermentans* P 140. Hamsters of group B were injected with *Mycoplasma fermentans* PG 18. Animals of group C were injected with Eaton strain of *Mycoplasma pneumoniae*, hamsters of group D were not injected with mycoplasmas. Hamsters of group E were injected with 0.1 ml of E media and group F (sham control) included animals that were stressed when they were puncture with a needle but they were not injected with mycoplasma or E media. All animals (controls and infected) received the same drugs (analgesics and anesthetics) so they were submitted to the same stress.

### Colonization of the respiratory tract and other organs

Three hamsters of each group (A to E) were sacrificed on days 1, 5, 10, 15, 25, 35 and 50 after the inoculation.

Two fragments of 1 cm^3^ of trachea, kidney, lung, heart, brain and spleen were cut. One fragment was cultured in E media and the other was processed in a histochinet for the histopathological study. A blood sample from each animal was cultured for mycoplasmas.

### Detection of mycoplasmas by culture

One fragment of each organ was cultured doing two tenfold dilutions in E media, each fragment was also cultured in agar blood plates to isolate aerobic bacteria. Broths were incubated at 37°C under aerobic conditions for 4 weeks or until the pH indicator of media turned yellow. A blind passage of broths on E plates was done on day 10. When the pH indicator of the media turned yellow a passage on E plates was done. Plates were incubated at 37°C under aerobic conditions for 4 weeks or until growth appeared. Positive cultures were identified as *Mycoplasma fermentans* or *Mycoplasma pneumoniae* by PCR.

### Detection of mycoplasmas by PCR

Polymerase chain reaction test was performed in all broth cultures (positive or negative) of different organs and in all organs extracted (direct detection) of infected animals and controls to detect the presence of *Mycoplasma fermentans*.

The oligonucleotide primers used for PCR assay were 5^′^-GGACTATTGTCTAAACAATTTCCC-3^′^ and 5^′^-GGTTATTCGATTTCTAAATCGCCT-3^′^, each primer is 24 bp long. This primer set flanks a 206-bp region in the *M. fermentans* genome [6]. We confirmed the specificity of these primers performing another PCR assay based on the amplification of the mycoplasmal 16S rRNA sequences published by van Kuppeveld FJ, et al. and we obtained a 272 bp PCR product, using the following primers: 5'-GAAGCCTTTCTTCGCTGGAG-3^′^ (forward primer) and 5'-ACAAAATCATTTCCTATTCTGTC-3' (reverse primer) [7].

The reaction mixture contained 50 mM KCl, 1.5 mM MgCl_2_, 10 mM Tris–HCl (pH 8.3), 0.2 mM of each deoxynucleotide triphosphate, 6 μM of each primer and 1 unit of AmpliTaq® in a total volume of 50 μl. The sample to be analyzed (5 μl) was always added last. A diluted lysate of *M. fermentans* PG-18 corresponding to 100 CCU and sterile water were used as positive and negative controls respectively. The amplification involved 40 cycles, each consisted of denaturation at 95°C for 25 s, primers annealing at 60°C for 60 s and extension at 72°C for 60 s. The amplified products were analyzed by electrophoresis in 2% agarose gels and visualized by UV light after ethidium bromide staining.

The oligonucleotide primers used for PCR detection of *Mycoplasma pneumoniae* were MP5-1 (GAAGCTTATGGTACAGGTTGG), MP5-2 (ATTACCATCCTTGTTGTAAGG) [8].

## Results and discussion

### Results

Three different doses of *Mycoplasma fermentans* P 140*, Mycoplasma fermentans* PG 18 and *Mycoplasma pneumoniae* Eaton (10^3^, 10^6^, 10^9^ CFU/ml) were tested in order to infect the hamsters. The first doses 10^3^ CFU/ml was not able to induce an interstitial pneumonia, 10^6^ CFU/ml induced it while 10^9^ CFU/ml induced a severe respiratory tract infection. Results were very similar using the three strains, so 10^6^ CFU/ml was chosen as the infective dose.

Table 1 shows positive cultures for mycoplasmas isolated from different organs and blood. Mycoplasmas were identified by PCR. Mycoplasmas were recovered from all the hamsters infected in at least one organ (trachea, lungs, heart, spleen, kidney, brain or blood) during the whole experiment. U frequency test was used to compare the recovery of the three strains from organs (Table 1); there were statistical differences between recovery of *Mycoplasma pneumoniae* and *Mycoplasma fermentans* PG 18 or P 140. *Mycoplasma pneumoniae* colonized all organs in a higher proportion than *Mycoplasma fermentans* (p<0.05).

**Table 1:** Recovery of mycoplasmas from organs and blood of experimentally infected hamsters

**Mycoplasma**	**Trachea**	**Right lung**	**Left lung**	**Heart**	**Spleen**	**Kidney**	**Brain**	**Blood**
*M. fermentans* P 140	11/21*	6/21	5/21	3/21	7/21	15/21	11/21	4/21
*M. fermentans* PG 18	2/21	6/21	3/21	6/21	11/21	13/21	4/21	2/21
*M. pneumoniae*	15/21	15/21	17/21	11/21	14/21	15/21	13/21	10/21

*Detected mycoplasmas in organs or blood/ total of infected hamsters.

*Mycoplasma fermentans* P 140 was detected by culture of organs along the experiment. Mycoplasmas colonized trachea during all the experiment, the bacteria also showed tropism to kidney and brain (Table 2).

**Table 2:** ***Mycoplasma fermentans *P 140 detection by culture and PCR in organs and blood of infected hamsters**

**Day**	**Trachea**	**Right lung**	**Left lung**	**Heart**	**Spleen**	**Kidney**	**Brain**	**Blood**	**Total**
1	1/3*	2/3	2/3	1/3	2/3	1/3	1/3	0/3	10/24
5	0/3	0/3	0/3	0/3	0/3	1/3	0/3	0/3	1/24
10	3/3	2/3	2/3	1/3	0/3	2/3	2/3	1/3	13/24
15	2/3	0/3	0/3	1/3	1/3	3/3	2/3	2/3	11/24
25	2/3	0/3	0/3	0/3	0/3	3/3	2/3	0/3	7/24
35	2/3	1/3	1/3	0/3	2/3	3/3	3/3	1/3	13/24
50	1/3	1/3	0/3	0/3	2/3	2/3	1/3	0/3	7/24
Total	11/21	6/21	5/21	3/21	7/21	15/21	11/21	4/21	

*Detected mycoplasmas in organs or blood/ total of infected hamsters.

*Mycoplasma fermentans* PG 18 colonized in a low rate the respiratory tract (trachea and lungs), but the bacteria showed tropism to spleen and kidney (Table 3).

**Table 3:** ***Mycoplasma fermentans *PG18 detection by culture and PCR in organs and blood of infected hamsters**

**Day**	**Trachea**	**Right lung**	**Left lung**	**Heart**	**Spleen**	**Kidney**	**Brain**	**Blood**	**Total**
1	0/3*	2/3	1/3	2/3	3/3	2/3	2/3	0/3	12/24
5	0/3	1/3	0/3	1/3	2/3	1/3	0/3	1/3	6/24
10	0/3	1/3	0/3	0/3	0/3	2/3	1/3	0/3	4/24
15	0/3	1/3	0/3	0/3	0/3	0/3	0/3	0/3	1/24
25	1/3	0/3	0/3	0/3	3/3	3/3	0/3	1/3	8/24
35	1/3	0/3	1/3	2/3	2/3	2/3	0/3	0/3	8/24
50	0/3	1/3	1/3	1/3	1/3	3/3	1/3	0/3	8/24
Total	2/21	6/21	3/21	6/21	11/21	13/21	4/21	2/21	

*Detected mycoplasmas in organs or blood/ total of infected hamsters.

Two hamsters were infected with the P 140 strain isolated from a lung of a hamster sacrificed on day ten after inoculation (second passage) another two hamsters were infected with the PG18 strain isolated from a lung of a hamster sacrificed on day ten after inoculation (second passage). There was no difference in the severity of the infection between animals infected with the first passage and the ones infected with the second passage.

*Mycoplasma pneumoniae* infected in a high rate the respiratory tract (trachea and lungs); it also showed tropism to heart, spleen, kidney and brain (Table 4).

**Table 4:** ***Mycoplasma pneumoniae *detection by culture and PCR in organs and blood of infected hamsters**

**Day**	**Trachea**	**Right lung**	**Left lung**	**Heart**	**Spleen**	**Kidney**	**Brain**	**Blood**	**Total**
1	3/3	2/3	3/3	2/3	3/3	3/3	3/3	2/3	21/24
5	2/3	3/3	2/3	1/3	2/3	3/3	2/3	1/3	16/24
10	1/3	2/3	1/3	2/3	0/3	0/3	1/3	0/3	7/24
15	3/3	3/3	3/3	2/3	3/3	3/3	2/3	0/3	19/24
25	0/3	1/3	3/3	1/3	2/3	2/3	2/3	2/3	13/24
35	1/3	2/3	2/3	3/3	2/3	2/3	1/3	2/3	15/24
50	0/3	2/3	3/3	0/3	2/3	2/3	2/3	3/3	14/24
Total	10/21	15/21	17/21	11/21	14/21	15/21	13/21	10/21	

*Detected mycoplasmas in organs or blood /total of infected hamsters.

### Macroscopic appearance of lungs

Lungs showed bilateral diffuse condensation similar to an interstitial pneumonia, few hemorrhagic areas without pleural effusion. *Mycoplasma pneumoniae* (positive control) and *Mycoplasma fermentans* P 140 showed a severe lesion. *Mycoplasma fermentans* PG 18 produced light lung lesions. (Illustration 1). Mycoplasmas were recovered from the infected lungs shown in the illustration 1.

### Histopathological study

*Mycoplasma fermentans* PG 18 and P 140 induced an interstitial pneumonia and tubular necrosis of the kidney during the whole experiment, in all lung samples damage increased slowly during the experiment until day 50. Renal damage was more severe in samples from animals infected with *Mycoplasma fermentans* PG 18.

Lung damage consisted of congestion of pulmonary capillaries and presence of infiltration of mononuclear cells in interstitial tissue (Table 5).

**Table 5:** Severity of histopathological damage observed in lungs during the experiment

**Day**	***M. fermentans*P 140**	***M. fermentans*PG 18**	***M. pneumoniae***
1	-	-	-
5	+	-	++
10	+++	+	+++
15	++	+	+++
25	++	+	++
35	++	++	++
50	++	++	++

Score:

- No damage in 100% of 10 fields observed.

+ Interstitial tissue infiltrated with inflammatory cells in less than 30% of 10 fields observed.

++ Interstitial tissue infiltrated with inflammatory cells in 30–60% of 10 fields observed.

+++ Interstitial tissue infiltrated with inflammatory cells in more than 60% of 10 fields observed.

*Mycoplasma fermentans* P 140 induced a more severe damage in lung than *Mycoplasma fermentans* PG 18 (Table 5). *Mycoplasma pneumoniae* produced an interstitial pneumonia during the whole experiment, renal damage was only observed from day 25 to 50.

**Figure 1:**
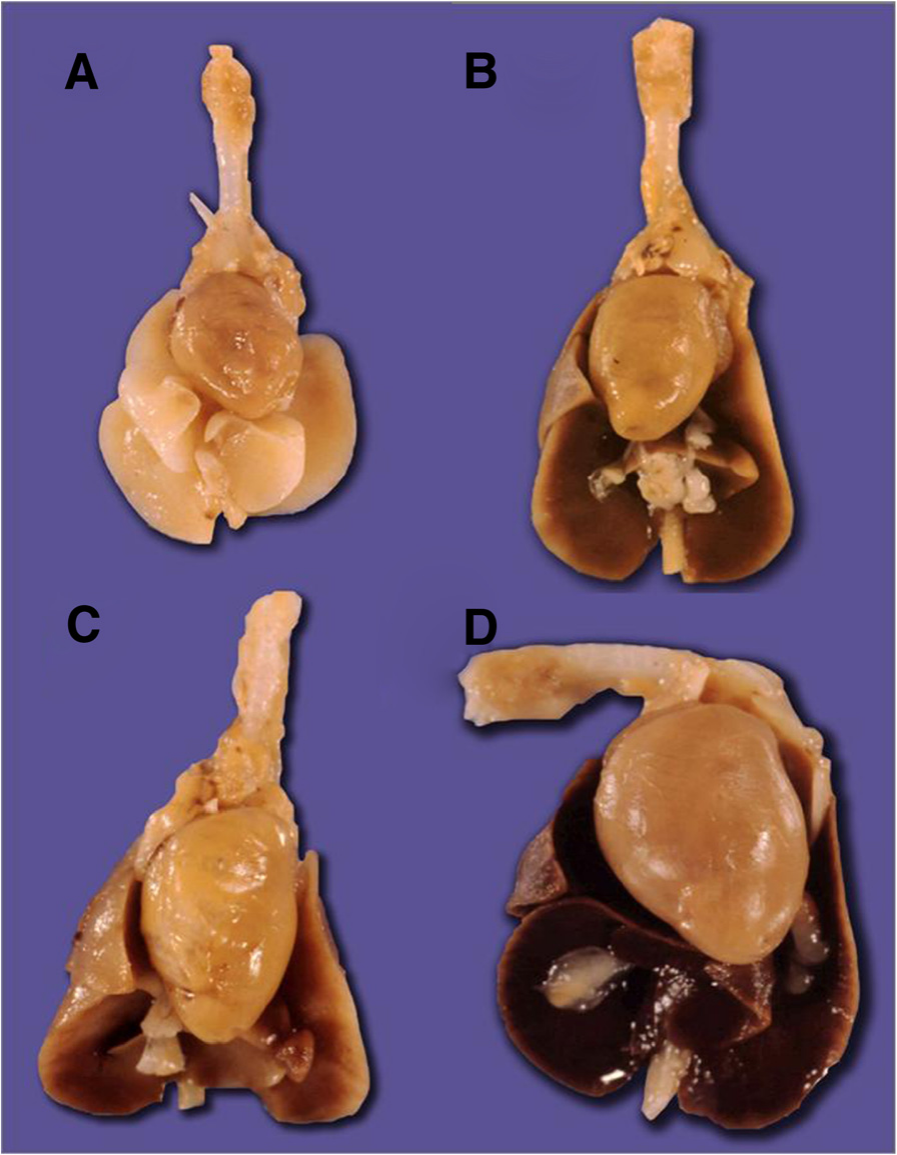
**Lung damage produced by mycoplasma infection.** Macroscopic appearance of lungs **A**. Control (uninfected hamster), **B**. hamster inoculated with *Mycoplasma pneumoniae*, **C**. hamster inoculated with *Mycoplasma fermentans* PG18 and **D**. hamster infected with *Mycoplasma fermentans* P 140. Animals infected (**B**, **C**, **D**) were injected with 0.1 ml of 10^6^ CFU/ml of a culture of mycoplasmas. Mycoplasmas were recovered from the infected lungs.

No histopathological damage was observed in heart, spleen and brain of hamsters infected with *Mycoplasma fermentans* P 140, PG 18 and *Mycoplasma pneumoniae*. No inflammatory response was observed in organs except in lungs of hamsters infected with mycoplasmas (Figure 1). Negative controls did not show damage in organs.

### Detection of mycoplasmas by pcr

All cultures (positive or negative) and samples from organs (direct detection) were searched for mycoplasmas by PCR.

Also the PCR products were sequenced by the Sanger method. We obtained a 99% to 100% homology with the *Mycoplasma fermentans* sequences reported at nucleotide blast service of the National Center for Biotechnology Information (NCBI).

There was a correlation between cultures and direct detection of mycoplasmas from organs. No mycoplasmas were detected from controls.

### Discussion

*Mycoplasma fermentans* isolated decades ago from the urogenital tract has been implicated in several diseases like rheumatoid arthritis and respiratory infections [9,10]. Interest in this organism has increased because of its possible role in the pathogenesis of rheumatoid arthritis [11-14].

The purpose of this study was to test the ability of *Mycoplasma fermentans* to induce a respiratory tract infection and migrate to several organs.

*Mycoplasma fermentans* P 140 was chosen because it produced a severe respiratory tract infection in an asthmatic patient and strain PG18 because it was isolated from the genitourinary tract and we supposed that it would not be able to induce a severe respiratory tract infection.

*Mycoplasma fermentans* P 140 was able to infect the respiratory tract and migrated to heart, spleen, kidney and brain probably through blood in experimentally infected hamsters. It was detected in a low amount in organs and blood on day 5 probably because of an effective immune response or because the bacteria is an intracellular organism. This strain was also able to persist in the respiratory tract and organs until day 50.

*Mycoplasma fermentans* PG18 induced a less severe respiratory tract infection even in animals inoculated with a strain that was recovered from an infected animal; bacteria migrated to different organs and persisted until day 50. It was not detected in organs or blood on day 15. This strain was originally isolated from the genitourinary tract probably the bacteria keeps its ability and preference to colonize the genitourinary tract and this is the reason why we isolated it in a higher percentage from the kidney (where it produced a severe histopathological damage) than the respiratory tract.

*Mycoplasma pneumoniae* was used as a positive control, this bacterium is one of the main causes of atypical pneumonia, it has been associated to pharyngitis, bronchitis, asthma, bronchiolitis, acute respiratory distress syndrome, acute chest syndrome, pericarditis, meningoencephalitis and arthritis [15-17]. *Mycoplasma pneumoniae* induced a severe respiratory tract infection in hamsters, migrated through blood and persisted during the whole experiment in the respiratory tract and different organs. Histopathological damage was only observed in lungs and trachea.

*Mycoplasma fermentans* P 140 was detected in a higher frequency from the respiratory tract than *Mycoplasma fermentans* PG18 (22 animals versus 11 animals). Severe damage in lungs of hamsters infected with *Mycoplasma fermentans* P 140 was observed. *Mycoplasma fermentans* PG18 induced mild damage in lungs (illustration 1). Severe damage appeared on day 10 in animals infected with P 140 strain while mild damage appeared on day 35 in lungs of hamsters infected with PG 18 strain. PG18 strain did not recover its virulence after one passage through an animal.

These facts suggested us that P 140 strain is more virulent than PG 18 strain.

All inoculated animals showed signs of infection (evidenced by recovery of the microorganism, detection of mycoplasma by PCR or histopathological damage). In some cases we detect the mycoplasma from all organs but in other cases from at least one organ. Mycoplasmas were not detected from controls.

This study showed the ability of *Mycoplasma fermentans* to persist in different organs for at least 50 days after inoculation, probably because they can evade the immune system and enter the cells where the antibodies and antibiotics cannot act and induce a chronic inflammation [18]. Intensive studies have been done in order to understand the strategy used by *Mycoplasma fermentans* to interact with host cells and evade the host protection system. The finding that some mycoplasmas can reside intracellularly helps to explain the development of chronic infectious diseases produced by mycoplasmas [19]. It has also been shown that mycoplasmas possess an impressive ability to maintain a surface architecture that is antigenically and functionally versatile and enable mycoplasmas to rapidly chance their antigenic characteristics [11]. These variable surface antigens undoubtedly contribute to the implication of *Mycoplasma fermentans* in chronic infections [11].

The ability of mycoplasmas to persist in organs and induce a chronic or even a latent infection may have implications in extrarrespiratory tract infections. Mycoplasmas can grow silently in close interaction with mammalian cells for a long period of time. However, prolonged interactions with mycoplasmas through a gradual and progressive course significantly affect many biologic properties of mammalian cells [20].

It has been shown that mycoplasmas need to be considered as a potential factor in the genesis of chronic inflammatory diseases. *Mycoplasma fermentans* serves as a stimulus for the production of several immune modulating cytokines and components of an inflammatory response. Mycoplasma may modulate inflammatory and immune processes within the lung as well as potentiate T cell dependent immunopathologies [18].

The role as pathogen of *Mycoplasma fermentans* has been controversial, it has been associated with genitourinary tract infections, rheumatic diseases and respiratory tract infections and it has also been considered as normal flora. Animal models of infection are particularly useful when the role as a pathogen of a microorganism is controversial. We choose Syrian hamsters to probe the ability of *Mycoplasma fermentans* to induce a respiratory tract infection because the histopathological changes produced by the infection resemble those that occur in natural human disease.

*Mycoplasma fermentans* produced pneumonia in hamsters and persisted in different organs for 50 days.

## Conclusions

The main contribution of this study was to show the ability of *Mycoplasma fermentans* to induce respiratory tract infection and its persistence in different organs in hamsters.

This study also showed the ability of *Mycoplasma fermentans* to migrate through blood, persist in several organs and help us answering the question about the persistence of viable mycoplasmas for weeks and the possibility that bacteria may induce chronic inflammation.

## Abbreviations

AIDS: Acquired immunodeficiency Syndrome; M. fermentans: Mycoplasma fermentans; M. pneumoniae: Mycoplasma pneumoniae; R. A: Rheumatoid arthritis; Mg: Milligrams; Kg: Kilograms; NCBI: National center for biotechnology information.

## Competing interests

The authors declare that they have no competing interests

## Authors’ contributions

AY conceived the study, participated in its design, performed PCR tests and help to draft the manuscript. AM performed the infection of animals and the microbiological study. TC performed the infection of animals and the microbiological study. FJGM performed the histopathological study. JAR performed the histopathological study and critically revised the manuscript. SG participated in the design of the study, revised critically the manuscript and helped to draft it. LC participated in the design and coordination of the study, performed the statistical analysis and helped to draft the manuscript. CG developed the animal model of infection. All authors read and approved the final manuscript.

## Authors’ information

AY is a Ph D on Microbiology is chief of the Microbiology Laboratory of the Faculty of Stomatology of the Universidad Autónoma de Puebla. JAR is a Ph D on Environment and Health and is a professor at the Universidad Autónoma de Puebla. SG is a Ph D on Microbiology, is a professor and researcher of the Instituto Politécnico Nacional. LC is a Ph D on Microbiology is a professor and researcher of the Universidad Autónoma de Puebla. AM, TC and FJGM are students of the Universidad Autónoma de Puebla. CG is a master on Microbiology, professor and researcher of the Universidad Autónoma de Puebla.
